# Rheumatoid interstitial lung disease presenting as cor pulmonale

**DOI:** 10.4103/0970-2113.71971

**Published:** 2010

**Authors:** Sourya Acharya, S. N. Mahajan, Samarth Shukla, S. K. Diwan, Pankaj Banode, Nirmesh Kothari

**Affiliations:** *Department of Medicine, J. N. Medical College, DMIMS, Sawangi (Meghe), Wardha, Maharashtra, India*; 1 *Department of Pathology, J. N. Medical College, DMIMS, Sawangi (Meghe), Wardha, Maharashtra, India*; 2 *Department of Radiology, J. N. Medical College, DMIMS, Sawangi (Meghe), Wardha, Maharashtra, India*

**Keywords:** Axial, cor pulmonale, polyarticular, pulmonary, rheumatoid arthritis, skeleton, synovitis

## Abstract

Rheumatiod arthritis (RA) is a multisystem connective tissue disorder. The predominant presentation is polyarticular, symmetric peripheral arthritis with relative sparing of axial skeleton. Inflammatory synovitis is the pathologic hallmark. Extra-articular manifestations of RA can involve several other organ systems and amongst them pulmonary manifestations occur commonly. We report a case of rheumatoid interstitial lung disease presenting as cor pulmonale.

## INTRODUCTION

Lung involvement in rheumatoid arthritis is common and usually presents with pleural disease or diffuse interstitial fibrosis.[1,2] Involvement of the small airways is a well-described but unusual manifestation, and involvement of the pulmonary vasculature is an extremely rare, and usually coincidental, finding at autopsy.[3,4] Few live case reports are available.[5,6]

Pulmonary fibrosis was first described in association with RA in the late 1940s.[7] It is now clear that the lungs and pleura manifest many different effects of RA.

## CASE REPORT

A 58-year-old female presented to us with history of dry cough on and off since one year, gradually progressive exertional dysnea since eight months. She was a diagnosed case of rheumatoid arthritis since 15 years. There was no h/o orthopnoea, PND, hemoptysis, and chest pain.

On clinical examination, pulse was 112/min, B.P was 114/68 mm of Hg, respiratory rate was 28/min with use of accessory muscles of respiration. Pallor was present. JVP was raised (10 cm of water), bilateral pitting edema was present up to knee joint.

**Figure 1:**
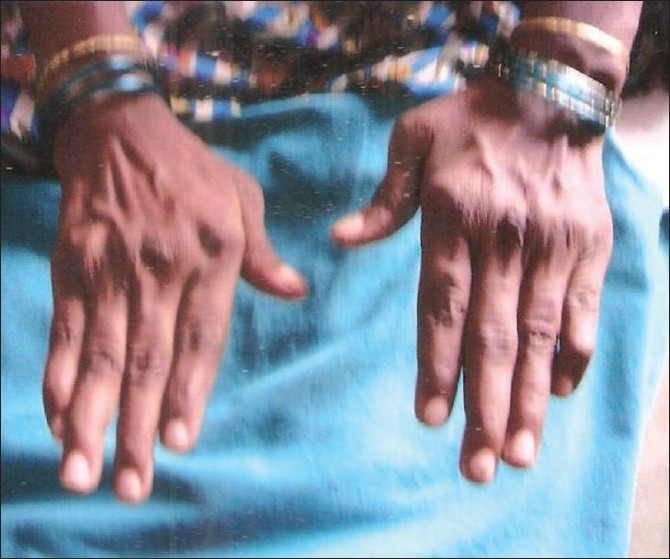
Showing classical deformities of rheumatoid arthritis

Stigmata of rheumatoid arthritis was present with characteristic deformities [Figure 1]. There were no palpable subcutaneous nodules, no purpura or neuropathy. CVS examination revealed diffuse right ventricular apex, parasternal heave of Grade 2, accentuated P2 at pulmonary area which was audible at apex. RS examination revealed bilateral diffuse coarse crepitations at lung bases. Per abdomen examination revealed soft, tender hepatomegaly. A provisional diagnosis of interstitial lung disease with cor pulmonale was kept.

**Figure 2:**
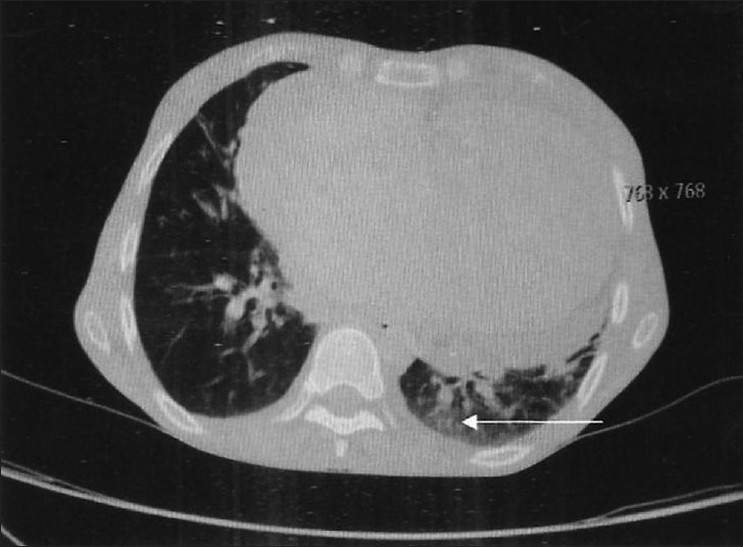
HRCT thorax showing ground glass opacity suggestive of interstitial lung disease

**Figure 3:**
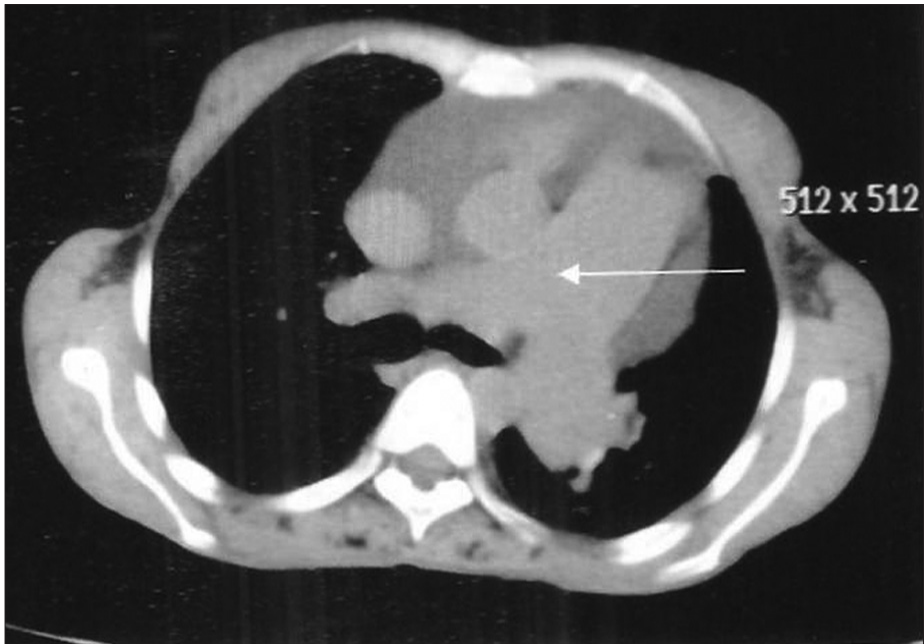
HRCT thorax showing dialated pulmonary artery

Investigations revealed Hb of 11.5gm%, TLC 4,200/mm^3^, DLC showed 58% polymorphs, 32% lymphocytes, 3% basophils, 5% monocytes, 2% eosinophils. ESR was 72 mm in first hour. RA factor was strongly positive. CXR showed cardiomegaly. PFT showed severe restriction (FEV1/VC)% Pred >99, FVC % Pred < 44. 2D ECHO revealed severe pulmonary arterial hypertension with a pulmonary artery systolic pressure of 56 mm of Hg, dialated right ventricle and functional TR. HRCT scan of thorax [Figures 2 and 3] revealed bilateral nodular infiltrates with ground glass opacification predominantly in the lower zones and dilatation of pulmonary artery.

Patient was started on oxygen therapy, Tab. Prednisolone 60 mg/day, Tab. Frusemide 40 mg/day and Tab. Digoxin 0.25 mg on alternate days, Tab salbutamol 4 mg in eight hourly interval and Formoterol/Budesonide (200micro. gm) nebulization every eight hours. After one week of treatment, patient reported moderate improvement in dysnea. At the time of discharge, her respiratory rate while resting in sitting position was 20/min, edema decreased though other parameters remained same. She was asked for follow-up after 15 days, but she has lost follow up.

## DISCUSSION

Little epidemiologic data are available on the occurrence of interstitial lung diseases (ILDs) in the general population. To describe the prevalence and incidence of ILDs, a population-based registry of patients with ILDs was established in Bernalillo County, New Mexico in October 1988. In this study, a total of 2,936 referrals were screened; 8.8% were prevalent cases and 6.9% were incident cases. Overall, the prevalence of ILDs was 20% higher in males (80.9 per 100,000) than in females (67.2 per 100,000). Similarly, the overall incidence of ILDs was slightly more common in males (31.5 per 100,000/year) than females (26.1 per 100,000/year). The estimated prevalence of preclinical or undiagnosed ILDs among all deaths was 1.8%. The most common incident diagnosed among both sexes were pulmonary fibrosis and idiopathic pulmonary fibrosis, together accounting for 46.2% of all ILD diagnoses in males and 44.2% in females. The study concluded that the occurrence of ILDs in the general population may be more common than previous estimates based on selected populations, and these disorders may frequently be unrecognized.[8]

The new ATS/ERS classification proposed the following clinicopathologic entities in order of relative frequency: idiopathic pulmonary fibrosis (IPF), nonspecific interstitial pneumonia (NSIP), cryptogenic organizing pneumonia (COP), acute interstitial pneumonia (AIP), respiratory bronchiololitis-associated interstitial lung disease (RB-ILD), desquamative interstitial pneumonia (DIP), and lymphoid interstitial pneumonia (LIP) [Table 1].

**Table 1:** Histological and clinical classification of idiopathic interstitial pneumonias*[9,10]

Histological patterns	Clinical, radiological, and pathological diagnoses
Usual interstitial pneumonia	Idiopathic pulmonary fibrosis/ cryptogenic fibrosing alveolitis
Nonspecific interstitial pneumonia	Nonspecific interstitial pneumonia (provisional)**
Organizing pneumonia	Cryptogenic organizing pneumonia***
Diffuse alveolar damage	Acute interstitial pneumonia
Respiratory bronchiolitis	Respiratory bronchiolitis interstitial lung disease
Desquamative interstitial pneumonia	Desquamative interstitial pneumonia
Lymphocytic interstitial pneumonia	Lymphocytic interstitial pneumonia

* Unclassifiable interstitial pneumonia: some cases are unclassifiable for a variety of reasons

** This group represents a heterogeneous group with poorly characterized clinical and radiological features that need further study

*** COP is the preferred term, but is synonymous with idiopathic bronchiolitis obliterans organizing pneumonia

Rheumatoid arthritis (RA) is a systemic inflammatory disease that can involve other tissues and organs as well as synovial joints. Extra articular manifestations of RA are well known. There is no agreed classification for these manifestations because of increased variability in criteria and definitions. Pulmonary manifestations are relatively common in 6-10% and are frequently present in early disease and are all related to worse outcomes measures of rheumatoid disease, in particular functional impairment and mortality.[11] Incidence of RA induced ILD is between 5 and 40%.[12] Clinically important ILD occurs in only 5-10% of patients of RA. The most common forms being usual interstitial pneumonias (UIP) and some degrees of non specific interstitial pneumonias (NSIP). Clinically significant cor pulmonale occurs in these subset of patients because of hypoxia-induced pulmonary vasoconstriction[13] [Table 2].

**Table 2:** Respiratory manifestations of rheumatoid arthritis are myriad

Pleural effusion/ fibrosis
Bronchopleural fistula
Pulmonary nodules and diffuse fibrosis
Caplan’s syndrome
Bronchiolitis obliterans
Recurrent infections
Eosinophilic pneumonia
Pulmonary hypertension
Shrinking lung
Bronchocentric granuloma
Cricoarytenoid obstruction
Bronchiectasis

Amongst all the respiratory conditions, fibrosis and pleural effusion are relatively common[14] Most commonly this occurs as a chronic, slowly progressive diffuse interstitial disease (UIP/NSIP). The process usually involves the lower zones. Unlike our case, it is more common in men, and contrary to our case the pulmonary involvement does not cause significant disability. Though there is no clear relationship of severity of rheumatoid arthritis and pulmonary fibrosis, evidence of pulmonary fibrosis in lung function testing is approximately 30 to 40% of all cases of RA.[15]

Pathologically, five different groups are identified on the basis of histological patterns namely rheumatoid pulmonary nodules, UIP, NSIP, BOOP (bronchiolitis obliterans organizing pneumonia), lymphoid hyperplasia, and cellular interstitial infiltrates.[16]

Clinically, the patient presents with cough with expectoration, hemoptysis, exertional dyspnea and nocturnal wheezing. Laboratory analysis shows rheumatoid factors, which are auto antibodies reactive with the Fc portion of Ig, found in more than two-thirds of adults with the disease; however, it is non-specific.[17] Pulmonary function tests show airway obstruction (decreased FEV1/VC), severe restriction (FVC decreased) or small airway disease (decreased FEF25-75)[18] DLco is a sensitive indicator of progression of lung disease. Plain radiograph of chest is non specific and shows pleural effusion/thickening, necrobiotic nodules, diffuse interstitial fibrosis, caplans syndrome and pulmonary hypertension.[19]

CT helps in identifying the lesions (reticulations, ground glass opacity, honey combing, lung nodules, consolidation, bronchiectasis, air trapping, pleural effusion/thickening, lymph node enlargement, pulmonary artery enlargement) and categorizing the findings into major CT patterns, namely usual interstitial pneumonia, non specific interstitial pneumonia, bronchiolitis and organizing pneumonia.[20]

However, the nature of the nodules cannot be determined from clinical or radiographic findings alone. A lung biopsy is needed to establish the diagnosis. Differential diagnosis of pulmonary nodules and underlying lung disorders in a patient with RA include mycobacterial infections, non-mycobacterial infections (nocardia, cryptococcus, histoplasma, aspergillus), neoplasms (bronchoalveolar carcinoma, multiple myeloma, lymphoma) and other collagen vascular diseases like SLE, systemic sclerosis, multiple connective tissue diseases, polyarteritis nodosa, dermatomyositis/ polymyositis.[21]

There is some evidence that the condition when associated with rheumatoid disease may stop progressing when patient is treated with high dose steroids. Non responders of corticosteroid treatment can be started on azathioprine or penicillamine. CT changes of a fine irregular type with significant atelectasis indicate mature fibrosis and poor response to therapy whereas a more fluffy or ground glass appearance usually indicates active inflammation and better response.[20]

## CONCLUSION

Pulmonary manifestations occur frequently in connective tissue diseases. There is no single manifestation of a particular disorder with considerable overlap between the different diseases, between associated pulmonary manifestations and between the same pulmonary conditions in absence of obvious collagen disease, there by, increasing the dilemma of diagnosis and treatment. It is prudent to have a suspicious index while treating patients with connective tissue diseases with a chronic respiratory problem and approach should be holistic including proper diagnosis of the disease.
